# The impact of digital start-up founders’ higher education on reaching equity investment milestones

**DOI:** 10.1007/s10961-017-9627-3

**Published:** 2017-10-03

**Authors:** Daniel Ratzinger, Kevin Amess, Andrew Greenman, Simon Mosey

**Affiliations:** 1 Haydn Green Institute for Innovation and Entrepreneurship, Nottingham University Business School, University of Nottingham, Nottingham, UK; 2 Nottingham University Business School, University of Nottingham, Nottingham, UK

**Keywords:** University qualifications, Human capital, Equity investments, Digital economy, L26, M13, J24, I23

## Abstract

This paper builds on human capital theory to assess the importance of formal education among graduate entrepreneurs. Using a sample of 4953 digital start-ups the paper evaluates the impact of start-up founding teams’ higher education on the probability of securing equity investment and subsequent exit for investors. The main findings are: (1), teams with a founder that has a technical education are less likely to remain self-financed and are more likely to secure equity investment and to exit, but the impact of technical education declines with higher level degrees, (2) teams with a founder that has doctoral level business education are less likely to remain self-financed and have a higher probability of securing equity investment, while undergraduate and postgraduate business education have no significant effect, and (3) teams with a founder that has an undergraduate general education (arts and humanities) are less likely to remain self-financed and are more likely to secure equity investment and exit while postgraduate and doctoral general education have no significant effect on securing equity investment and exit. The findings enhance our understanding of factors that influence digital start-ups achieving equity milestones by showing the heterogeneous influence of different types of higher education, and therefore human capital, on new ventures achieving equity milestones. The results suggest that researchers and policy-makers should extend their consideration of universities entrepreneurial activity to include the development of human capital.

## Introduction

High growth start-ups are considered to be a significant driver of economic growth and researchers and policy-makers alike are concerned with understanding their emergence and growth (Grilli and Murtinu 2014; Lerner 2010; Mason and Brown 2014). Digital start-ups are of particular interest as they are expected “to deliver more value and wealth to more consumers and citizens more broadly than any economic development since the Industrial Revolution” (Dean et al. 2012, p. 5). Fundamental to the creation and growth of digital start-ups is the successful exploitation of new knowledge (Audretsch et al. 2016; De Laurentis 2006). A significant repository of that knowledge is to be found within university departments of computer science and business schools (Kollmann 2006). Yet, the process by which such knowledge is transferred to digital start-ups remains underexplored.

Universities, through their research and teaching, are recognized to be vital contributors to the growth of technology industries (Belitski and Desai 2016), with particular attention being paid to their entrepreneurial activity. Although entrepreneurial practices in both teaching and research (Etzkowitz 2003), have been recognized to have a positive economic impact (Guerrero et al. 2015), studies have historically focused upon the commercialization of intellectual property created from university research activities as a primary mechanism for knowledge transfer (Siegel and Wright 2015). These activities, however, represent a “distinct minority of all academic interactions with external organizations” (Hughes and Kitson 2012, p. 734) and the broader role of entrepreneurial universities is being recognized, with an increasing focus upon other entrepreneurial individuals beside academics (Audretsch 2014). For instance, the deployment of creative problems skills by graduates, gained through the teaching practices of the traditional faculties of a university have been argued to be vital for innovation and economic development (Philpott et al. 2011). More recently, attention has therefore shifted towards the knowledge transfer contribution of university students, particularly those who show an interest in entrepreneurship as a postgraduate career option (Fayolle and Gailly 2015). Studies have considered the economic impact and employment contribution that graduates make through creating start-ups (Siegel and Wright 2015), yet how the knowledge is transferred from universities to these graduate entrepreneurs has received rather less attention (Wright 2014; Nelson 2012).

We propose that a focus upon the human capital that universities contribute to graduate entrepreneurs could therefore yield new insights. Education is generally argued to be amongst the most crucial investments in human capital (Becker 2009) and the factor that also has the most direct effect (Schultz 1961). Through teaching, universities could therefore be seen as major contributors to the skillsets of start-up founders. Human capital is widely recognized in entrepreneurship research (Marvel 2013) and has also been linked to economic growth (Calcagnini et al. 2016), but few studies have focused on the impact of universities on the performance of start-ups through the human capital they contribute (Nielsen 2015). This may be in part due to the contradictory findings across different studies. Some authors argue that human capital provided through education provides a consistent and positive effect upon start-up performance (Oakey 2012). Others suggest that there may be an upper limit to the benefits of education and that even customized programs, such as entrepreneurship degrees, may increase entrepreneurial engagement but do not show any discernible impact upon venture performance (Davidsson and Honig 2003). We aim to reconcile this debate by considering digital start-ups, a type of venture that are arguably the most reliant upon knowledge based assets (Dean et al. 2012), and unpick the relative effects of different types of higher education upon the performance of their graduate founders.

A challenge in considering the performance of such early stage and private businesses is the absence of traditional financial information. One approach is to adopt investment milestones as proxy performance measures (Chang 2004). This could be via angel investments (Kerr et al. 2014) or venture capital investments (Colombo and Grilli 2010; Mason and Brown 2014). Although it is generally very difficult for start-ups to secure such equity investments (Van Osnabrugge 2000), those that do so are more likely to achieve growth (Colombo and Grilli 2010).

From an investment point of view, successful start-up growth trajectories can be therefore captured by observing the securing of an equity investment, followed by an acquisition or initial public offering in order to liquidize the equity holders’ investments (DeTienne 2010; Hegde and Tumlinson 2014).

Regardless of the performance measures used, prior research on digital start-ups has tended to neglect the impact of human capital due to a focus upon firm level attributes (Hayter et al. 2016). We address this gap through assessing the impact of higher education on digital technology entrepreneurs’ ability to reach the equity investment milestones of funding and exit. With a focus on universities as educators, this study therefore explores their impact on the digital economy in terms of their human capital contribution to start-up founding teams through formal higher education.

In the following section, we review contemporary work within human capital theory to build hypotheses relating specific types of higher education to digital start-up performance. Next, we describe the methodology used and then present a summary of our findings. We conclude this article with a discussion of the findings and provide suggestions for further research.

## Human capital theory

With knowledge recognized to be a core asset of start-ups in the digital economy (Kollmann 2006) human capital theory is an appropriate approach to evaluate the impact of higher education upon that knowledge. A human capital approach is widely utilized in entrepreneurship research (Marvel 2013) and has also been used to explain economic growth (Calcagnini et al. 2016). Human capital theory assumes that an individual’s performance outcome is related to their skill and knowledge levels (Martin et al. 2013) and that increased performance and productivity levels can be expected with increased human capital (Schultz 1961). By contrast, personal traits are not considered as human capital because they cannot be transferred or developed over time. Within entrepreneurship, human capital has been identified as a much more significant performance indicator than personal traits (Wright et al. 2007). Entrepreneurs with higher levels of human capital have been found to be more likely to identify entrepreneurial opportunities (Davidsson and Honig 2003; Shane 2000). In addition, it has been proposed that human capital has a positive influence on venture performance, although it has also been noted that related empirical evidence is inconclusive (Davidsson and Honig 2003; Marvel and Lumpkin 2007).

For instance, Eesley and Roberts (2012) disentangled human capital into the two concepts of innate talent and the accumulation of entrepreneurial experience. They highlighted that rather than there being a general positive impact of human capital upon venture performance, using this finer grained distinction show that the relative importance of experience versus talent changes with the context (i.e., when the current market or technology is familiar, experience dominates and vice versa). We therefore propose to take a similar approach to untangle the effect of human capital upon digital start-ups performance. If we focus upon the specific types of human capital provided by higher education then we should be able to tease out the impact of education in different disciplines and at different levels of study, when controlling for other factors.

In more general studies, formal education has generally been found to have an influence upon engaging in entrepreneurship, but when it comes to the success of subsequent entrepreneurial activities, when controlling for previous start-up experience, education was not seen to have any significant impact (Davidsson and Honig 2003). It was concluded, that “even the most specific type of explicit human capital, formal education as provided by business classes, only succeeded in increasing the pace of gestation activities, not in affecting critical outcomes” (Davidsson and Honig 2003, p. 322).

By contrast, research focusing upon the digital economy (Kollmann 2006) and the technology industry overall (Oakey 2012), have shown human capital in the form of business and technical knowledge to increase start-up performance.

A confounding effect which may reconcile these inconsistent findings is that many start-ups consist of multiple founders who work as a team. While research investigating the human component of entrepreneurship initially focused on individual entrepreneurs, with the maturing of the research area a shift towards entrepreneurial teams is observed (Forbes et al. 2006). Teams may confer benefits, especially within knowledge-based industries, and it has been argued that entrepreneurial teams, rather than individual entrepreneurs, more commonly drive the venture creation process. Moreover, start-up investors in particular have noted concerns regarding the skill sets and expertise held by the whole founding team (Kamm et al. 1990). For example, while specific multidisciplinary skill sets of computer science and business skills required for an effective digital start-up in the digital economy (Kollmann 2006) may be difficult to obtain on an individual level, such mixed skill sets can be acquired within a founding team (Stuetzer et al. 2012).

For this study, a team is therefore defined as “two or more individuals who jointly establish a business in which they have an equity (financial) interest” (Kamm et al. 1990, p. 7) and, to enable a comparative analysis, single founders are also considered as a one-person team in our analysis. As human capital has an influence on performance at an individual level (Martin et al. 2013), the composition of the founding team is arguably influential upon the performance of the start-up. Although it has been suggested that diversity in a team can have a positive impact on performance, homogeneous teams with a shared cognition are commonly seen in academic spinouts, leading to suboptimal outcomes (Vanaelst et al. 2006). The underperformance of teams with members that have similar backgrounds has also been confirmed in an earlier study on high-tech spin-offs (Clarysse and Moray 2004) and Ruef et al. (2003) concluded that, although a rational team formation based on the highest achievable outcome could be expected in the specific case of competitive start-up founding teams, team formation is commonly driven by familiarity and trust rather than functional performance.

These counterfactual findings suggest that graduate founders are most likely to build successful ventures if they have a skillset that is matched to the needs of the specific industry sector (Kollmann 2006; Oakey 2012). Therefore, for the digital sector we propose that founders who have studied business or technical education at university (such as computer science or engineering), when controlling for other factors, should outperform those that have not:

### **Hypothesis 1**

Start-up founders with higher business education, or higher technical education, have a higher probability of reaching investment milestones.

Building upon this more granular approach, human capital theory has also been criticized due to common assumption that “more human capital is always better” and that over-investments are therefore not being considered (Davidsson and Honig 2003, p. 305). Indeed, as discussed by Marvel and Lumpkin (2007), certain types of human capital may not be as desirable as others for entrepreneurship. For instance, formal education and deep knowledge in an area have been found to be more significant for enabling radical innovation than broad knowledge across a number of areas. However, the inverse relationship has been found to enable incremental innovation (Marvel and Lumpkin 2007).

In this vein, we propose that a specific human capital configuration that consists of formal higher education in technical and business related disciplines thereby meeting the specific knowledge needs of the digital economy (Kollmann 2006) should have a higher impact on the probability of reaching investment milestones than more general education, which we define as higher education in non-technical and non-business disciplines (such as the arts, humanities and social sciences):

### **Hypothesis 2a**

Higher business education has a greater impact on the probability of reaching investment milestones than more general higher education.

### **Hypothesis 2b**

Higher technical education has a greater impact on the probability of reaching investment milestones than more general higher education.

Previous studies have relied on the number of years of any type of formal education as a measure of human capital (Marvel and Lumpkin 2007), although this approach has been challenged by Klomp (2013) as it reduces the multi-faceted concept of human capital to a single dimension (Klomp 2013). It has been highlighted, that research needs to move beyond such single measures for human capital (McGuirk et al. 2015). Particularly at a higher education level, the multi-faceted nature becomes evident with an increase in degree levels that shows a shift from practice to research. While the undergraduate and postgraduate level are mainly focused on generally applicable learning outcomes such as problem solving skills and communication, the most advanced level of doctorates is more generally concerned with developing those skills for specific application within the academic community (Bologna Working Group on Qualifications Frameworks 2005). This would suggest a non-linear relationship between education level and venture performance, with the possibility of potential upper limits to the value of human capital gained through formal education for venture performance (Davidsson and Honig 2003). This is contested by Oakey (2012), who suggests consistent and linear increases in performance with concomitant increases in technical ability and business acumen. Consequently, we propose the following hypothesis to assess the impact of specific higher education degree levels upon venture performance:

### **Hypothesis 3**

Increasing university degree levels lead to increasing probability of reaching investment milestones.

## Research methodology

### Data source

We use a novel data source that is crowd-sourced, operated by CrunchBase and published under the Creative Commons Attribution License (www.crunchbase.com). CrunchBase’s mission is “to make information about the startup world available to everyone and maintainable by anyone” and therefore its data is not just created and maintained by AOL employees (particularly by people working for TechCrunch.com) but also by the general public. Anyone who accepts the terms and conditions can register on CrunchBase to get access to the dataset. In the context of this project, participants are companies and people whose information is stored in the CrunchBase database.

CrunchBase offers a unique opportunity to explore technology entrepreneurship on a large scale with records on more than 270,000 people that are linked to more than 220,000 companies. It has also been demonstrated that CrunchBase data is particularly reliable when it comes to financial data (Block and Sandner 2011). The crowd-sourced information, which is moderated before being accepted, is a comprehensive source of data on start-ups, entrepreneurs, and key milestones (e.g. investments and achieved exits). This enables a between-group analysis of graduate and non-graduate founders that have secured an equity investment or that have exited their start-up.

### Data sample

The data sample used in this study has been retrieved from CrunchBase between 1 May 2014 and 10 May 2014. It has subsequently been filtered for start-ups that have at least one valid person with a job role containing one of the following three keywords to capture start-up founders only: owner, founder, founding. While CrunchBase provides various information about people and companies, some information that is vital for this study was missing. The gender of each founder is determined with an algorithm utilizing a database containing 134,138 first names that was developed for this study, with data openly published by various national statistics agencies. Based on the total number of occurrences of a particular name for a specific gender, a gender probability measure is calculated for every first name found in the dataset.

**Fig. 1:**
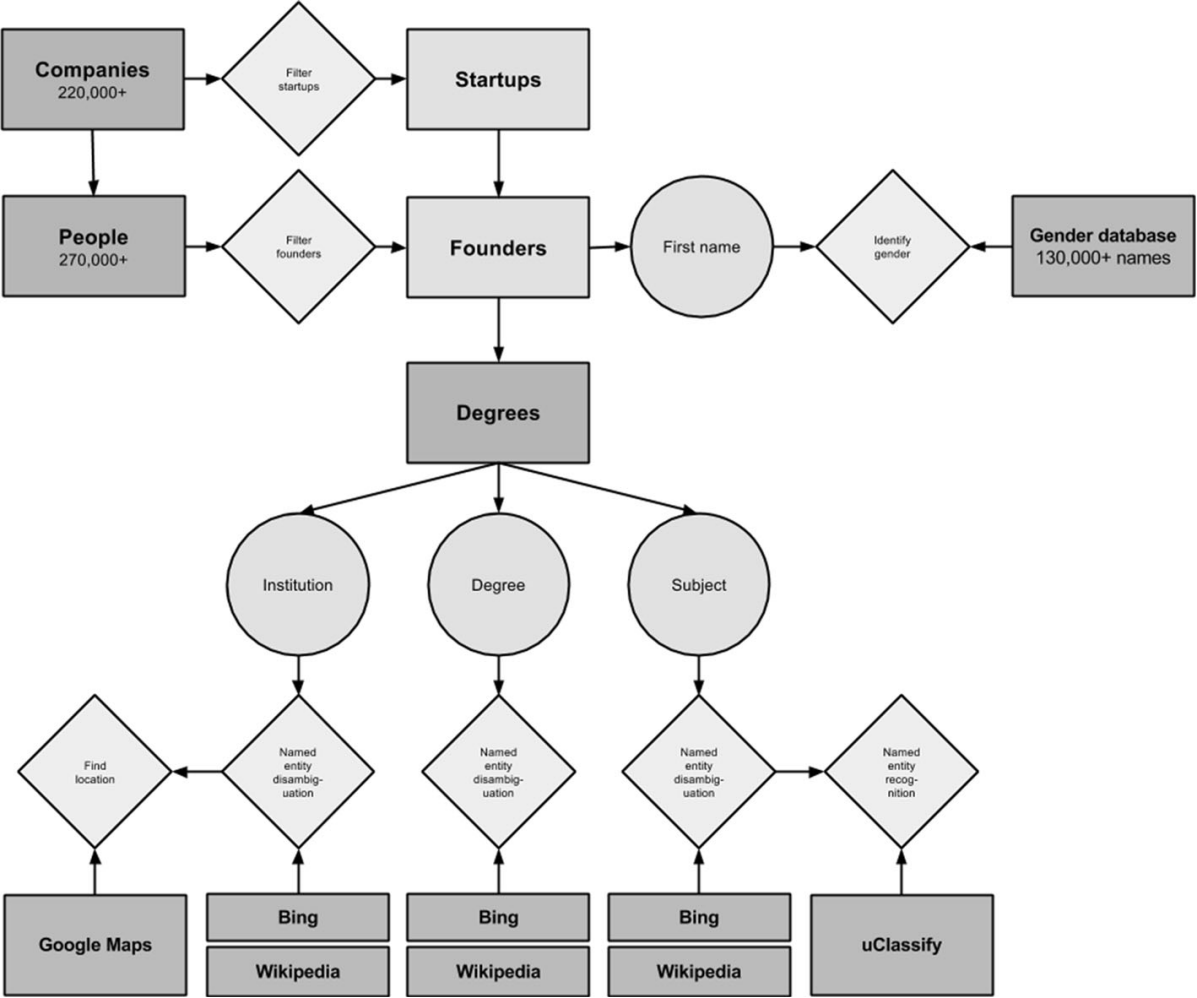
Data model entity relationship diagram

Furthermore, each founder’s degree(s) are recorded in a semi-structured manner. While degree subject, degree level, and institution are stored separately, the information in these fields is unstructured text that required processing prior so that quantitative techniques could be employed. This required a mixture of named entity disambiguation and named entity recognition analysis in order to identify the information held within data points and to further categorize the information. Algorithms for pre-processing the data points are developed that link to the following additional data sources, particularly in order to overcome named entity recognition as well as named entity disambiguation challenges: Microsoft Bing, Wikipedia and Google Maps as knowledge bases and uClassify.com for natural language processing. Figure 1 shows the data model entity relationship.

We obtain information on more than 220,000 start-ups stored on Crunchbase. We then filter and clean the data in the following ways. First, we selected start-ups founded in the last 25 years. Second, digital start-ups were selected from the following categories on Crunchbase: advertising; e-commerce; enterprise software and services; games and video; hardware; mobile; network hosting; search; security; software; web; messaging; and analytics. Third, the data points are cleaned and categorized. Fourth, only start-ups where the founder provided complete information on their Crunchbase profile and a distinctively identified degree subject are used in the analysis. As a consequence of these processes 4953 digital start-ups are included in our data set. The characteristics of the final sample are extensively tested by comparing it with existing studies, including data from the Global Entrepreneurship Monitor, gender comparisons (Coleman and Robb 2009) and a pilot study conducted previously (Ratzinger et al. 2013). Overall, on a descriptive level the CrunchBase data reveals similar results to other studies when investigating higher education levels of start-up founders.

Although extensive work has been conducted to test the robustness and reliability of the data sample, the limitations need to be recognized. Issues have been controlled to the best of the authors’ knowledge, but the fact remains that the original data sample is crowd-sourced and is focused on many other data points in addition to human capital. In addition, the choice of control variables was limited by the data source.

### Empirical strategy

We aim to test the hypotheses stated in the prior section and determine and quantify the impact of higher education qualifications on digital start-ups achieving two investment milestones: funding and exit. These investment milestones are binary outcomes that are ordered and therefore lend themselves to being analyzed using an ordered probit model, which assumes a preferred order of outcomes (Halabí and Lussier 2014). The estimating equation is specified as follows:

$$ {\text{s}}_{f} = {\beta}_{0} {\text{EDUCATION}}_{f} + {\beta}_{1} {\text{Z}}_{f} + {\text{u}}_{i} $$

where subscript *f* (*f* = 1, …, *F*) denotes the *f*th start-up and *F* denotes the total number of start-ups in the cross section; s represents the dependent variable that identifies the investment milestones that identify three different states for start-ups on an ordinal scale: self-sustaining (0); funding (1); and exit (2). EDUCATION represents the following dichotomous education variables: higher technical education; higher business education; and general higher education. For the more detailed analysis required for testing Hypothesis 3, each of the education variables is further distinguished between the different degree levels: undergraduate; masters; and doctorate. Z represents a vector of control variables capturing the following start-up characteristics and founder demographics: start-up age; number of cofounders; and founders’ gender. In addition, a scale measures the proportion of founders in a team who have obtained their degree from the same university. Founders prior start-up experience is captured in a variable with the following scale: 0 (self-sustained/non-funded start-up), 1 (funded start-up) to 2 (exited start-up).

### Descriptive statistics

Table 1 provides the descriptive statistics of the overall sample as well as within each skill set group.

**Table 1:** Founder team characteristics and education profile

	Overall	Higher education		
		Technical	Business	General
Investment milestones				
Self-sustained	53.6%	45.4%	49.5%	46.8%
Funded	40.1%	45.3%	44.0%	45.9%
Exited	6.2%	9.4%	6.5%	7.3%
Higher education				
Technical	34.5%	–	37.2%	28.9%
Business	27.8%	30.0%	–	32.2%
General	7.5%	6.3%	8.6%	–
Graduate founders	55.8%	–	–	–
Undergraduate	44.1%	81.2%	80.7%	84.6%
Postgraduate	21.6%	43.7%	44.4%	33.5%
Doctorate	5.5%	13.5%	5.9%	17.6%
Start-up				
Average business age	5.94	6.25	5.45	5.70
Average number of cofounders	2.01	2.35	2.28	2.42
Founders				
All male	72.7%	69.4%	73.2%	66.5%
All female	4.1%	2.9%	3.6%	8.6%
Mixed gender	2.6%	3.6%	4.0%	5.4%
Unknown	20.6%	24.1%	19.3%	19.5%
Social capital				
Avg. cofounders attending same university	–	23.8%	23.9%	21.1%
Entrepreneurial experience	19.3%	22.6%	19.7%	21.9%
Self-sustained start-up	11.2%	11.5%	10.4%	11.1%
Funded start-up	4.6%	5.7%	4.9%	5.1%
Exited start-up	3.6%	5.4%	4.4%	5.7%
Observations	4953	1710	1379	370

As can be seen from Table 1, the average number of cofounders in a start-up team is 2.01; however, 44.09% of all start-ups have only got a single founder. More than half of all founding teams (55.8%) have at least one founder who is educated to a degree level and overall about 34.5% of founding teams have founders with technical and 27.8% with business skills at a degree level. When analyzing the skill sets of teams that have reached specific investment milestones, it can be seen that a slightly larger proportion of teams with founders with technical degrees have been able to secure funding or exit their business compared to founders with business education (54.6 vs. 50.5%). A larger proportion of teams dominated by business graduates also have technical graduates compared to the proportion of teams dominated by technical graduates that also have business graduates (37.2 vs. 30.0%). However, overall the difference between teams with technical graduates and teams with business graduates appears fairly minor.

Table 2 shows a cross-tabulation of the proportion of teams with different levels and investment milestones. It shows the proportion of teams with graduate founders with self-sustaining businesses is significantly smaller (48.7 vs. 59.9%) while the proportion of teams with funded or exited businesses is larger than compared to teams without any graduate founders. In total, more than half of all degree educated founding teams have secured funding or have managed to exit their business. Furthermore, it can be seen that the proportions of teams that have secured funding is decreasing with lower degree levels, with 56.1% of all doctorates, 47.0% of all postgraduates and 43.4% of all undergraduates.

**Table 2:** Cross-tabulation of degree level and investment milestones

	No degree (%)	Degree (%)	Degree level		
			Undergraduate (%)	Postgraduate (%)	Doctorate (%)
Investment milestones					
Self-sustained	59.9	48.7	48.2	43.4	33.9
Funded	36.0	43.4	43.4	47.0	56.1
Exited	4.1	7.9	8.3	9.6	10.0

## Results

Ordered probit estimates of the determinants of digital start-ups achieving investment milestones are reported in Table 3. The results show that technical education, business education, general education are statistically significant determinants of digital start-ups achieving investment milestones. The control variables that are found to be statistically significant determinants of digital start-ups achieving investment milestones are: gender and cofounders attending the same university. The results are obtained from estimation of ordered probit regressions and the estimated marginal effects, conditional on the mean, for each variable used in the regressions.

**Table 3:** Determinants of achieving investment milestones (ordered probit regression)

Investment milestone	Coefficient	z-statistic
Higher education		
Technical	0.23***	6.26
Business	0.09**	2.44
General	0.14**	2.19
Start-up		
Age	0.05***	12.70
Number of cofounders	0.10***	5.92
Gender		
Female	−0.01	−0.17
Mixed	0.11	1.11
Unknown	0.15***	3.71
Social Capital		
Cofounders attending same university	0.23**	2.57
Entrepreneurial experience		
Self-sustained start-up	−0.42***	−6.66
Funded start-up	0.15*	1.81
Exited start-up	0.36***	3.94
Number of obs	4953	
Wald chi^2^	420.67	
Prob > chi^2^	0.00	
Pseudo R^2^	0.05	

***,**,* Significance at the 1, 5, and 10 level, respectively

Reported in Table 4 are the marginal effects, which quantify the impact of determinants on achieving investment milestones. The marginal effects figures represent the impact of the probabilities of outcomes. Here, we focus on those variables relevant to hypotheses 1 and 2. The probability of being self-sustained is 9, 4, and 5 percentage points lower for co-founders with technical, business, and general degrees, respectively. In contrast the probability of being funded is 7, 3, and 4 percentage points higher if the co-founders have a technical, business, and general degree, respectively. Finally, the probability of exiting is 3, 1, and 2 percentage points higher for co-founders with technical, business, and general degrees, respectively.

**Table 4:** Estimated marginal effects of determinants on each equity milestone

	Self-sustained		Funded		Exited	
	M. E.	z-statistic	M. E.	z-statistic	M. E.	z-statistic
Higher education						
Technical	−0.09***	−6.28	0.07***	6.37	0.03***	5.74
Business	−0.04**	−2.44	0.03**	2.47	0.01**	2.36
General	−0.05**	−2.19	0.04**	2.27	0.02**	2.00
Start-up						
Age	−0.02***	−12.68	0.02***	12.42	0.01***	10.83
Number of cofounders	−0.04***	−5.92	0.03***	5.85	0.01***	5.79
Gender						
Female	0.01	0.17	0.00	−0.17	0.00	−0.17
Mixed	−0.04	−1.11	0.03	1.14	0.01	1.02
Unknown	−0.06***	−3.71	0.04***	3.78	0.02***	3.46
Social capital						
Cofounders attending same university	−0.09**	−2.57	0.07**	2.56	0.02***	2.59
Entrepreneurial experience						
Self-sustained start-up	0.16***	7.04	−0.13***	−6.62	−0.03***	−8.30
Funded start-up	−0.06*	−1.82	0.04*	1.90	0.02	1.64
Exited start-up	−0.14***	−4.06	0.09***	4.76	0.05***	3.18

*M. E.* marginal effect

***, **, and * indicate significance at the 1, 5, and 10% level, respectively

We now provide brief account of the control variables. Co-founders attending the same university, our proxy for social capital, are less likely to be self-sustained and an increased likelihood of being funded and of exiting. Founding teams prior experience of being self-sustained increases the likelihood of the current venture being self-sustained by 16 percentage points while reducing the likelihood of achieving funding and exit milestones. In contrast, prior experience of achieving funding and exit milestones decreases the current venture’s likelihood of being self-sustained but increases its likelihood of achieving funding and exit milestones. Both, start-up age and number of cofounders have shown to positively influence the probabilities of funding or exiting.

In order to test Hypothesis 3 an ordered probit model containing specific degree level attributes is estimated and the results are reported in Table 5. Results show that different levels of technical higher education (i.e. undergraduate, postgraduate and doctorate degrees) are all statistically significant determinants of achieving investment milestones. In contrast, not all levels of business and general higher education impact on investment milestones. Doctorate level business education is significant at the 5% level while undergraduate business education is significant at the 10% level.

**Table 5:** The impact of specific higher education on reaching investment milestones (ordered probit regressions)

Investment milestone	Coefficient	z-statistic
Technical education		
Undergraduate level	0.19***	4.52
Postgraduate level	0.23***	3.99
Doctorate level	0.21***	2.58
Unknown degree level	−0.02	−0.17
Business education		
Undergraduate level	0.05	1.05
Postgraduate level	0.10*	1.89
Doctorate level	0.60**	2.36
Unknown degree level	0.20*	1.96
General education		
Undergraduate level	0.19***	2.59
Postgraduate level	0.09	0.60
Doctorate level	0.16	1.02
Unknown degree level	−0.09	−0.54
Start-up		
Age	0.05***	12.49
Number of cofounders	0.10***	5.81
Gender		
Female	−0.02	−0.20
Mixed	0.10	0.94
Unknown	0.13***	3.23
Social Capital		
Cofounders attending same university	0.18**	2.02
Entrepreneurial experience		
Self-sustained start-up	−0.42***	−6.62
Funded start-up	0.16*	1.87
Exited start-up	0.35***	3.78
Number of obs	4953	
Wald chi^2^	464.85	
Prob > chi^2^	0.00	
Pseudo R^2^	0.06	

***,**,* Significance at the 1, 5, and 10% level, respectively

In order to determine the influence on various specific milestones, marginal effects have been calculated and are reported in Table 6. We first comment on the impact of technical education. Undergraduate, postgraduate and doctoral technical education all have a negative impact, significant at the 1% level, on the likelihood of a venture being financially self-sustained. In contrast, the different levels of technical education all have a statistically significant positive impact on the probability of achieving funding and exit milestones. The likelihood of being funded increases by 6 percentage points for postgraduate and doctoral technical education while the probability of achieving exit increases by 3 percentage points for postgraduate education. The other impacts of technical education are more modest.

**Table 6:** Marginal effects of specific higher education on achieving equity milestones

Marginal effects	Self-sustained		Funded		Exited	
	M. E.	z-statistic	M. E.	z-statistic	M. E.	z-statistic
Technical education						
Undergraduate level	−0.07***	−4.52	0.05***	4.64	0.02***	4.14
Postgraduate level	−0.09***	−4.01	0.06***	4.26	0.03***	3.46
Doctorate level	−0.08***	−2.59	0.06***	2.76	0.02**	2.25
Unknown degree level	0.01	0.17	−0.01	−0.17	0.00	−0.17
Business education						
Undergraduate level	−0.02	−1.05	0.01	1.06	0.01	1.03
Postgraduate level	−0.04*	−1.88	0.03*	1.93	0.01*	1.77
Doctorate level	−0.23***	−2.59	0.13***	4.31	0.10*	1.67
Unknown degree level	−0.08**	−1.97	0.05**	2.10	0.02*	1.71
General education						
Undergraduate level	−0.08***	−2.60	0.05***	2.75	0.02**	2.27
Postgraduate level	−0.04	−0.60	0.03	0.62	0.01	0.56
Doctorate level	−0.06	−1.02	0.05	1.08	0.02	0.91
Unknown degree level	0.04	0.55	−0.03	−0.53	−0.01	−0.59
Start-up						
Age	−0.02***	−12.47	0.02***	12.23	0.01***	10.64
Number of cofounders	−0.04***	−5.81	0.03***	5.75	0.01***	5.69
Gender						
Female	0.01	0.20	−0.01	−0.20	0.00	−0.20
Mixed	−0.04	−0.94	0.03	0.97	0.01	0.88
Unknown	−0.05***	−3.23	0.04***	3.29	0.01***	3.04
Social capital						
Cofounders attending same university	−0.07**	−2.02	0.05**	2.01	0.02**	2.03
Entrepreneurial experience						
Self-sustained start-up	0.16***	7.00	−0.13***	−6.59	−0.03***	−8.23
Funded start-up	−0.06*	−1.88	0.04**	1.97	0.02*	1.69
Exited start-up	−0.14***	−3.88	0.09***	4.50	0.05***	3.06

*M. E.* marginal effect

***,**, * Significance at the 1, 5, and 10% level, respectively

The marginal effects in Table 6 shows that not all levels of business education have a statistically significant effect on the probability of achieving investment milestones. Doctoral business education reduces the likelihood of being self-sustained by 23 percentage points and the probability of being funded by 13 percentage points. No level of business education had an impact on achieving the exit milestone better than the 10% level of significance.

Table 6 shows that only undergraduate general education had a statistically significant impact on achieving investment milestones. The probability of being self-sustained is 8 percentage points lower while the probability of achieving funding and exit increases by 5 percentage points and 2 percentage points, respectively.

## Discussion

Although Davidsson and Honig (2003) did not observe a relationship between business education and the performance of ventures, this study suggests that the probability of securing funding increases by 3 percentage points if someone in the cofounding team has obtained a business degree. While the specific content of the studied subjects is unclear in this study, the results are more in line with the results presented by Tan and Ng (2006) who have identified entrepreneurship education as a factor that increases an entrepreneur’s confidence to participate in high growth businesses.

The expectations that start-ups in the digital space benefit most from technical education, which can be seen as industry specific human capital, has been confirmed. Technical education increases the probability of receiving funding by 7 percentage points and the probability of exiting a business by 3 percentage points. The importance of such industry specific human capital is also reflected in the increase of probabilities on each technical degree level. With both, technical and business education being significant factors in increasing the probabilities of reaching investment milestones, the importance of balanced skill sets, as highlighted by Stuetzer et al. (2012), can be confirmed. Hypothesis 1 can therefore be accepted.

When discussing balanced skill sets, not only industry specific human capital, in the form of technical education and entrepreneurship specific human capital in the form of business education (e.g. Kollmann 2006; Oakey 2012), should be considered. As can be seen in this specific industry, although all start-ups can be expected to have a technical element in their business model in common, opportunities that are being exploited may be found in completely unrelated industries. Besides the technical element, the range of industries that digital start-ups operate within varies highly and while this study has insufficient data to distinguish between each specific type of education that may be relevant, we capture general education as all non-technical and non-business subjects. The contribution of such general higher education in addition to the expected business and technical knowledge needed in a digital start-up is an unexpected finding, with general higher education showing an increase in the probability of receiving funding by 4 percentage points and in the probability of exiting the business by 2 percentage points. This is remarkable in that it shows a higher impact than business education. In addition, although higher technical education demonstrates the greatest impact on the probability of reaching both investment milestones, general higher education shows an impact that is greater by 1 percentage point than higher business education for each investment milestone. Although some teams may not possess the recommended technical and business skills (e.g. Kollmann 2006; Oakey 2012), it could be assumed that this advanced knowledge in a specific field, and the transferable skills obtained during their studies at a degree level (Bologna Working Group on Qualifications Frameworks 2005), give them a competitive advantage in exploiting an opportunity that they have identified (Davidsson and Honig 2003; Marvel and Lumpkin 2007). Hypothesis 2a is therefore rejected while hypothesis 2b is accepted, which suggests that for digital start-ups, technical education is preeminent (Marvel and Lumpkin 2007).

The analysis of the separate degree levels has revealed that higher levels do not necessarily lead to higher performance. While some of the degree levels have not revealed any significant results, technical education at a doctorate level increases the probability of exiting a start-up by 1 percentage point less than at a postgraduate level. While higher technical education had a greater impact than higher business education overall, by far the largest impact on a specific degree level has been observed for business education at a doctorate level. This further emphasizes the importance of distinguishing between higher degree levels as opposed to measuring education in number of years or as a dichotomous variable. We therefore contribute to the debate on upper limits of human capital by highlighting the value of moving beyond a single measure (McGuirk et al. 2015) to capture the multiple dimensions of human capital (Klomp 2013), and of education in general (Bologna Working Group on Qualifications Frameworks 2005). Hypothesis 3 is consequently rejected, which confirms the importance of specific human capital configurations as opposed to overall high levels of human capital (Marvel and Lumpkin 2007) in the digital sector.

The results of this analysis contribute to the commonly raised but under-investigated issue of over-investments in human capital (Davidsson and Honig 2003). While the investment in human capital by means of education and training are academically well understood (Becker 2009), the role of universities is being increasingly challenged in the digital sector (e.g. Nathan et al. 2012). However, the results of this study suggest that universities as educators potentially play a significant role. Higher education increases the probabilities of receiving funding or exiting a business significantly. This study has illustrated the universities’ impact by linking their primary mission of teaching to their more contemporary mission of economic development (Etzkowitz and Leydesdorff 2000). With higher educated founding teams indicating increased probabilities of reaching milestones closely related to high growth venture performance, a correlation to economic impact is evident. This is notable as teaching is an activity that has not traditionally been evaluated in economic terms (Philpott et al. 2011).

## Conclusions

This study makes a novel contribution to the human capital literature, by analyzing the impact of higher education on start-ups in an industry sector of growing importance. Although the digital sector is increasingly questioning the role of universities (e.g. Nathan et al. 2012), the results have indicated that universities have a considerable influence upon digital start-ups performance. Through making a finer grained examination of the effect of higher education we show that increased formal business and technical education within founding teams increases the probability of reaching investment milestones for digital start-ups. In addition, and counterintuitively, general higher education in the arts, humanities or social sciences also provide performance benefits.

By focusing upon the context of the digital economy, this research also contributes by examining the impact of specific human capital (Jayawarna et al. 2011), in the form of education developed at university and broken down by subject area, on the distinct performance measures of probability and timing of reaching start-up investment milestones (Unger et al. 2011). Crucially, this research moves beyond a single measure for human capital (McGuirk et al. 2015) by examining the multi-dimensional nature (Klomp 2013) of the education variable. Consequently, other than previously thought (Criaco et al. 2014; Jayawarna et al. 2011; Millán et al. 2014; Oakey 2012; Schultz 1961), the findings of this research indicate that human capital, in the form of higher education, has a heterogeneous influence on high growth start-up performance in the form of reaching equity investment milestones. We therefore reconcile the debate surrounding the potential negative effects of overinvestments in human capital (Davidsson and Honig 2003). Although technical degrees are seen to be valuable for graduate founders, overinvestment in technical education produces lower returns in the digital sector.

The analysis has been conducted on a relatively large dataset that was readily available. In line with previous suggestions (Gao et al. 2016), the value in using such a dataset has been demonstrated with the findings of this research. Therefore, attention has also been drawn to the potential of using existing datasets in entrepreneurship research, although it needs to be recognized that the results are limited by the nature of the crowd sourced dataset. In addition, the statistical analysis in this study has been conducted on each estimation coefficient individually. Further research should consider the analysis of combined factors in order to investigate the effect of a combination of human capital factors.

## Managerial implications

For policy makers and practitioners, the results can act as guidance for understanding the impact of human capital on start-up performance. Attention is also drawn to the importance of including students from a variety of disciplines in entrepreneurship education programs such as the Summer of Student Innovation organized by Jisc (2015), which is aimed at all students in UK higher and further education, or the Digital Futures Young Entrepreneurs Scheme organized by the University of Nottingham, which is aimed at postgraduate and postdoctorate researchers (Digital Economy Network 2015). Practitioners should recognize that higher education has an impact on digital start-ups reaching major investment milestones. Of particular note is the finding that the impact varies based on studied subject area and degree level. Broadly speaking, practitioners, such as investors, should be actively seeking teams with mixed skillsets, as they show the strongest relative performance in reaching major start-up investment milestones. Whereas the existing literature is predominantly focused on inculcating industry-relevant skills in technology and business (Kollmann 2006, p. 334), practitioners need to take notice that general higher education in subjects other than technology and business can also have a positive impact. Generally, as a practitioner, it is therefore worth examining the educational background of digital start-up founders and its applicability to the specific start-up in more detail.

Considering educational background, practitioners should also be aware of the potential pitfalls that seem to be associated with deep subject knowledge, such as that obtained through a doctorate degree. The heterogeneous nature of human capital observed in this research project can lead to adverse effects on the performance in terms of reaching investment milestones.

With clear economic impact, indirectly resulting from higher education, policy makers should also rethink the way the output of the entrepreneurial university is being measured. This research has taken a first step to demonstrate the viability of using previously underutilized open datasets for quantifying indirect academic entrepreneurship.

## Limitations and future research

With the analyses conducted in this study, the probabilities of achieving specific outcomes have been investigated. Whilst an increase in probabilities to reach specific stages can certainly be understood as a positive sign, timing is arguably of equal importance (Kerr et al. 2014). However, at this stage, the speed at which those outcomes are achieved remains unknown, which could be of interest to policy makers concerned about economic growth as well as private equity investors. Therefore, to add another dimension to this research, further work could focus on the timing of reaching specific investment milestones. This research has taken a first step towards investigating the impact of the start-up founding teams’ human capital configuration over time (Muñoz-Bullon et al. 2015), and the findings of this exploratory research can act as the basis for future investigations. However, it needs to be highlighted that this research generalized higher education in the data analysis, and consequently did not measure the effect of specific universities on digital start-ups. We hope that the findings of this research will help guide future studies to address such limitations.
